# Hemoadsorption in the critically ill—Final results of the International CytoSorb Registry

**DOI:** 10.1371/journal.pone.0274315

**Published:** 2022-10-25

**Authors:** Fatime Hawchar, Dana Tomescu, Karl Träger, Dominik Joskowiak, Klaus Kogelmann, Jens Soukup, Singrun Friesecke, David Jacob, Jan Gummert, Andreas Faltlhauser, Filippo Aucella, Martijn van Tellingen, Manu L. N. G. Malbrain, Ralph Bogdanski, Günter Weiss, Andreas Herbrich, Stefan Utzolino, Axel Nierhaus, Andreas Baumann, Andreas Hartjes, Dietrich Henzler, Evgeny Grigoryev, Harald Fritz, Friedhelm Bach, Stefan Schröder, Andreas Weyland, Udo Gottschaldt, Matthias Menzel, Olivier Zachariae, Radovan Novak, Jernej Berden, Hendrik Haake, Michael Quintel, Stephan Kloesel, Andreas Kortgen, Stephanie Stecher, Patricia Torti, Frieder Nestler, Markus Nitsch, Detlef Olboeter, Philip Muck, Michael Findeisen, Diane Bitzinger, Jens Kraßler, Martin Benad, Martin Schott, Ulrike Schumacher, Zsolt Molnar, Frank Martin Brunkhorst

**Affiliations:** 1 Department of Anesthesiology and Intensive Care, University of Szeged, Szeged, Hungary; 2 Department of Anaesthesia and Critical Care, Fundeni Clinical Institute, Bucharest, Romania; 3 Carol Davila University of Medicine and Pharmacy Bucharest, Bucharest, Romania; 4 Kardioanasthesiologie, Universitätsklinikum Ulm, Ulm, Germany; 5 Universitätsklinikum der LMU München, Herzchirurgische Klinik und Poliklinik, Munich, Germany; 6 Klinik für Anästhesiologie und Intensivmedizin, Hans-Susemihl-Krankenhaus GmbH, Emden, Germany; 7 Klinik für Anästhesiologie, Intensivtherapie und Palliativmedizin, Carl-Thiem-Klinikum Cottbus, Cottbus, Germany; 8 Klinik und Poliklinik für Innere Medizin B, Universitätsmedizin Greifswald, Greifswald, Germany; 9 Universitätsklinik für Allgemein-, Viszeral- und Gefäßchirurgie, Universitätsklinikum Magdeburg, Magdeburg, Germany; 10 Herz- und Diabeteszentrum NRW, Klinische Studien Chirurgie, Bad Oeynhausen, Germany; 11 Kliniken Nordoberpfalz AG, Klinikum Weiden, Weiden, Germany; 12 Research Hospital "Casa Sollievo della Sofferenza" Medical Science, Nephrology and Dialysis Unit, San Giovanni, Rotondo, Italy; 13 Ziekenhuis De Tjongerschans, Heerenveen, Netherlands; 14 First Department of Anaesthesiology and Intensive Therapy, Medical University of Lublin, Lublin, Poland; 15 Medical Data Management, Medaman, Geel, Belgium; 16 International Fluid Academy, Lovenjoel, Belgium; 17 Klinik für Anästhesiologie, AG Hämodynamik, Klinikumrechts der Isar TU München, München, Germany; 18 Krankenhaus Hietzing, Wiener Krankenanstaltenverbund, A, Wien, Austria; 19 Klinik für Anästhesiologie, Intensivmedizin und Schmerztherapie, Klinikum Region Hannover Nordstadt, Hannover, Germany; 20 Universitätsklinikum Freiburg, Abteilung Allgemein- und Viszeralchirurgie, Freiburg, Germany; 21 Klinik für Intensivmedizin, Universitätsklinikum Hamburg- Eppendorf, Hamburg, Germany; 22 Klinik für Anästhesie, Intensiv-, Palliativ- und Schmerzmedizin, Berufsgenossensch Uniklinik Bergmannsheil, Bochum, Germany; 23 Krankenhaus BHS Ried im Innkreis, Ried im Innkreis, Austria; 24 Klinikum Herford, UK Anästhesie, Intensivmedizin, Rettungsmedizin, Schmerztherapie, Herford, Germany; 25 Institute for Complex Issues of Cardiovascular Diseases, Kemerovo, Russian Federation; 26 Krankenhaus Martha Maria Halle Klinik für Anaesthesiologie und Intensivmedizin, Halle, Germany; 27 Klinik für Anästhesiologie, Intensiv-, Notfallmedizin, Transfusionsmedizin und Schmerztherapie, Evangelisches Krankenhaus Bielefeld, Bielefeld, Germany; 28 Krankenhaus Düren gem. GmbH, Klinik für Anästhesiologie, Intensivmedizin, Notfallmedizin und Schmerztherapie, Düren, Germany; 29 Universitätsklinik für Anästhesiologie/Intensiv-/Notfallmedizin/Schmerztherapie, Klinikum Oldenburg GmbH, Carl von Ossietzky Universität, Oldenburg, Germany; 30 Heinrich Braun Klinikum Zwickau, Zwickau, Germany; 31 Klinikum Wolfsburg, Klinik für Anästhesie und Intensivmedizin, Wolfsburg, Germany; 32 Klinikum Oberlausitzer Bergland GmbH, Zittau, Germany; 33 University Medical Centre Ljubljana, Dep. Of Internal medicine, ICU, Ljubljana, Slovenia; 34 Klinik für Kardiologie und Intensivmedizin, Kliniken Maria Hilf GmbH, Mönchengladbach, Germany; 35 Zentrum Anästhesiologie, Rettungs-und Intensivmedizin, Universitätsklinikum Göttingen, Göttingen, Germany; 36 GPR Klinikum Rüsselsheim, Abteilung Anästhesie, Rüsselsheim, Germany; 37 Universitätsklinikum Jena, Klinik für Anästhesiologie und Intensivmedizin, Jena, Germany; 38 Medizinische Klinik und Poliklinik II, Klinikum der Universität München, München, Germany; 39 Rianimazione Ospedale U. Parini, S.C. Anestesia e Rianimazione, Aosta, Italy; 40 Kliniken Erlabrunn gGmbH, Erlabrunn, Germany; 41 Klinik für Anästhesie, Intensiv-, Notfallmedizin und Schmerztherapie, Krankenhaus St. Elisabeth und St. Barbara, Halle, Germany; 42 Krankenhaus Herzberg, Elbe-Elster-Klinikum GmbH, Herzberg, Germany; 43 Universitätsklinikum Schleswig-Holstein, Lübeck, Germany; 44 Klinik für Pneumologie, Gastroenterologie, Internistische Intensiv- und Beatmungsmedizin, Städtisches Klinikum München GmbH, Klinikum Harlaching, Munich, Germany; 45 Universitätsklinikum Regensburg, Klinik für Anästhesiologie, Regensburg, Germany; 46 Fachkrankenhaus Coswig, Klinik für Anästhesiologie und Intensivmedizin, Coswig, Germany; 47 Bodden Kliniken Ribnitz Damgarten, Ribnitz Damgarten, Germany; 48 Diakovere Friederikenstift, Hannover, Germany; 49 Center for Clinical Studies Jena (ZKS), Jena University Hospital, Jena, Germany; 50 Doctoral School of Multidisciplinary Medical Sciences, University of Szeged, Szeged, Hungary; 51 Institute for Translational Medicine, School of Medicine, University of Pécs, Pécs, Hungary; 52 Department of Anaesthesiology and Intensive Therapy, Poznan University of Medical Sciences, Poznan, Poland; 53 Department of Anaesthesiology and Intensive Therapy, Semmelweis University, Budapest, Hungary; 54 Center for Sepsis Control and Care (CSCC), Jena University Hospital, Jena, Germany; 55 Department of Anaesthesiology and Intensive Care Medicine, Jena University Hospital, Jena, Germany; IRCCS Policlinico S.Donato, ITALY

## Abstract

The aim of the current paper is to summarize the results of the International CytoSorb Registry. Data were collected on patients of the intensive care unit. The primary endpoint was actual in-hospital mortality compared to the mortality predicted by APACHE II score. The main secondary endpoints were SOFA scores, inflammatory biomarkers and overall evaluation of the general condition. 1434 patients were enrolled. Indications for hemoadsorption were sepsis/septic shock (N = 936); cardiac surgery perioperatively (N = 172); cardiac surgery postoperatively (N = 67) and “other” reasons (N = 259). APACHE-II-predicted mortality was 62.0±24.8%, whereas observed hospital mortality was 50.1%. Overall SOFA scores did not change but cardiovascular and pulmonary SOFA scores decreased by 0.4 [-0.5;-0.3] and -0.2 [-0.3;-0.2] points, respectively. Serum procalcitonin and C-reactive protein levels showed significant reduction: -15.4 [-19.6;-11.17] ng/mL; -17,52 [-70;44] mg/L, respectively. In the septic cohort PCT and IL-6 also showed significant reduction: -18.2 [-23.6;-12.8] ng/mL; -2.6 [-3.0;-2.2] pg/mL, respectively. Evaluation of the overall effect: minimal improvement (22%), much improvement (22%) and very much improvement (10%), no change observed (30%) and deterioration (4%). There was no significant difference in the primary outcome of mortality, but there were improvements in cardiovascular and pulmonary SOFA scores and a reduction in PCT, CRP and IL-6 levels.

**Trial registration:** ClinicalTrials.gov Identifier: NCT02312024 (retrospectively registered).

## Background

Hyperinflammation is a common feature in critically ill patients, which can be provoked by infectious or non-infectious insults [1]. While this immune response is necessary to recover from the disease or after major surgery or trauma, it can also become dysregulated evolving into uncontrolled hyperinflammation [2, 3]. This so-called cytokine storm can have serious adverse effects mainly due to the mass release of vasoactive substances that can cause severe vasodilatation and hemodynamic instability, damage the endothelium and the glycocalyx leading to capillary leakage and interstitial fluid accumulation, and potentially impair vital organ functions [4].

This has led to the assumption that bulk removal of cytokines, endotoxins, tissue degradation proteins and other mediators of inflammation via extracorporeal blood purification may prove beneficial [5, 6]. Several forms of blood purification approaches have been tested over the last decades with contradicting results [7–9].

One of the most recent developments is CytoSorb^®^ (CytoSorbents, USA). This is a 300 mL container filled with biocompatible, highly porous polystyrene divinylbenzene beads that form a large surface of about 45,000 m^2^ and adsorbing hydrophobic molecules up to approximately 55 kDa. As most cytokines fall within this range the device is potentially capable of eliminating toxic substances rapidly from the blood. The product can be used in combination with renal replacement therapy, cardiopulmonary bypass, Extra Corporeal Membrane Oxygenation (ECMO) or on its own as hemoperfusion [10].

Despite its relatively short history–the product was registered in 2011 –, the number of patients treated with CytoSorb is continuously growing and the number of single treatments worldwide is approaching 80,000 (information from CytoSorbents). The same holds true for the International CytoSorb Registry that was created in May 2015 and has been independently led by the Centre for Clinical Studies at the Jena University Hospital, Germany. The number of centres participating, and the number of entries is also continuously growing. The first paper on the 3^rd^ interim analysis on 198 patients was published in 2017 [11] and since then the number of entries has just recently crossed the 1000 mark, as confirmed by the Centre for Clinical Studies at the Jena University Hospital, Germany.

The purpose of the current paper is to summarize the results of the International CytoSorb Registry.

## Methods

The study protocol was registered on ClinicalTrials.gov (Identifier: NCT02312024) on the 9th of December 2014. Since then, any institute that uses CytoSorb voluntarily registered via www.cytosorb-registry.org, after which a start package was sent out. Once ethics approval from the corresponding research and development organizations and informed consent was obtained data collection took place. The inclusion criteria were: use of CytoSorb adsorber, age ≥18 years and signed informed consent. There were no exclusion criteria.

### Ethics approval and consent to participate

This study protocol has been submitted to and approved by the Institutional Review Board of the Faculty of Medicine at Friedrich Schiller University, Jena that acts as the IRB in charge for Germany (NCT: NCT02312024). All German ethic committees involved are informed about the participation of centers in their area of responsibility and the decision of the IRB in charge. In centers from outside Germany, approval of the local ethics commission in charge is obtained and all national regulations are adhered to. The list of all the ethics committee/institutional review boards that approved our study is the following: National (Germany) International:Ethik-Kommission der Universitätsmedizin Göttingen (Ethics commitee—first votum), Ärztekammer Berlin Ärztekammer Niedersachsen, Ärztekammer Nordrhein, Ärztekammer Sachsen-Anhalt, Ethik-Kommission an der Medizinischen Fakultät der Universität Rostock, Ethik-Kommission Bayrische Landesärztekammer, Ethikkommission bei der Ärztekammer Schleswig-Holstein, Ethik-Kommission bei der Landesärztekammer Hessen, Ethik-Kommission der Albert-Ludwigs-Universität Freiburg, Ethik-Kommission der Ärztekammer Hamburg, Ethik-Kommission der Ärztekammer Westfalen- Lippe und der Westfälischen Wilhelms-Universität Münster, Ethik-Kommission der Fakultät für Medizin der Technischen Universität München, Ethik-Kommission der Friedrich Schiller Universität Jena Ethikkommission der LMU München, Ethikkommission der Medizinischen Fakultät der Heinrich-Heine-Universität Düsseldorf, Ethik-Kommission der Medizinischen Fakultät der Ruhr-Universität Bochum(RUB), Ethik-Kommission der Medizinischen Fakultät der Ruhr-Universität Bochum(RUB), Sitz Bad Oeynhausen, Ethik-Kommission der Medizinischen Hochschule Hannover, Ethik-Kommission der Otto-von-Guericke-Universität Magdeburg, Ethik-Kommission der Universität Ulm, Ethik-Kommission der Universitätsmedizin Göttingen, Ethikkommission der Universitätsmedizin Greifswald, Ethik-Kommission Universität Lübeck, Ethik-Kommission der Medizinischen Fakultät der Martin-Luther-Universität Halle Wittenberg- Halle, Landesärztekammer Brandenburg, Sächsische Landesärztekammer Dresden, Thüringer Landesärztekammer.

International: Scientific Research Institut for Complex Issues of Cardiovascular Diseases—Local Ethical Committee, Kemerovo, Russia; Semmelweis University Regional and Institutional Committee of Science and Research, Ethics, Budapest; Regional and Institutional Ethics Committee, Clinical Center, University of Debrecen; Regional Human Biomedical Research Ethics Committee Szeged; Komisija Republike Slovenije za medicinsko etiko; Ethikkommission der Stadt Wien; Consiliul de Etica, Institutul Clinic Fundeni, Bucharest; Ethikkommission Krankenhaus Ried,Barmherzige Schwestern; Ethikkommission der Stadt Wien; Comitato Etico Azienda U.S.L. Valle d’Aosta; Il Comitato Etico presso la Fondazione Casa Sollievo della Sofferenza di San Giovanni Rotondo nella; Medisch Ethische Commissie Ziekenhuis Tjongerschans Heerenveen; Commissie Medische Ethiek Brussel Comitato Etico Palermo 2; Comitato Etico ASL Lecce; Comitato Etico Regionale Per La Basilicata, Matera; Comitato Indipendente di Etica Medica, Brindisi.

Responsible party: Jena University Hospital. Patients were included after written informed consent.

The data are not publicly available due to privacy or ethical restrictions, since data contain potentially identifying or sensitive patient information. The data that support the findings of this study are available on request to the Center of Clinical Studies at Jena University Hospital (JUH), Salvador-Allende-Platz 27, 07747 Jena, Germany. E-mail: zks@med.uni-jena.

### Patients

Fundamentally there were four main categories of patients for which data collection forms had been specified. Those who were treated for 1) sepsis, septic shock (Sepsis group) [12], 2) patients who underwent cardiac surgery with the use of cardiopulmonary bypass (CPB) and treated with CytoSorb intraoperatively (Pre-emptive group), 3) those who were treated after cardiac surgery in the postoperative period on the intensive care unit (ICU) (Postoperative group) and 4) those who were treated for other indications than the previous three (Other group).

### Interventions

There were no specific interventions apart from hemoadsorption therapy that was recommended to be used according to the user’s guide provided by CytoSorbents. The cartridge needs to be incorporated into an extracorporeal circuit and used on its own as hemoperfusion, or together with renal replacement therapy, cardiopulmonary bypass or ECMO. Length of treatment by one adsorber is recommended to be used for up to 24 hours.

### Data collection and management

Detailed description of the data collection and management was discussed previously [11]. In brief, data collection was performed on electronic case report forms (eCRF). Data were recorded at 4 time points: at baseline (demographics, indications, severity scores, recorded within 24 hours before hemoadsorption), physiological and laboratory data collected right before the start of therapy (T1) and up to 24 hours after the last hemoadsorption treatment (T2) and finally follow up on discharge from the hospital. Data was collected by a dedicated staff. Training of the staff was performed by the CytoSorb Registry project manager and knowledge was distributed locally by the physicians responsible for the project in every hospital. Data was stored on the servers of the Center for Clinical Studies at Jena University Hospital using the OpenClinica study management software.

### Outcomes

The Primary endpoint was defined as the difference between predicted mortality by APACHE II score and actual mortality after intervention during hospital stay, as recommended for registries for evaluating patient outcomes [13].

Secondary endpoints were:

- Organ function as indicated by a change in SOFA score before and after treatment (T2-T1)
- Concentration changes (T2-T1) of biomarkers: IL-6, C-reactive protein (CRP), procalcitonin (PCT), myoglobin, free hemoglobin
- Length of ICU and hospital stay (days)
- Duration of mechanical ventilation (days)
- Duration of renal replacement therapy (days)
- Duration of vasopressor therapy (days)
- Subjective assessment of the change of the patients’ condition by the attending physician using a scale from “very much improved” to “very much worse” (for details please see the results).

Adverse events: one of the objectives of the Registry was to record complications related to the use of the device.

### Statistics

All available data were displayed using appropriate descriptive statistics. This includes at least number of non-missing values, number of missing values, mean, standard deviation, minimum, quartiles, median, interquartile ranges and maximum for metric data and frequencies for categorial data. Where appropriate, change to baseline for metric data were evaluated and analysed or shift tables for frequencies were displayed.

For the APACHE II score, mortality rates were evaluated according Knaus et al. [14]. Mortality rates (predicted and true) were compared by use of a logistic regression model analogous to the one developed by Knaus et al.; the significance level was pre-set at Alpha = 0.05. SAPS II Score and predicted mortality rates were analysed analogously. In addition to the predicted mortality according Le Gall [15], predicted mortality according to Haaland [16] was analyzed.

Change of the SOFA scores was analyzed by using t-test as well as a linear model with baseline level (Exam 1) as covariate.

### Results and discussion

**Fig 1:**
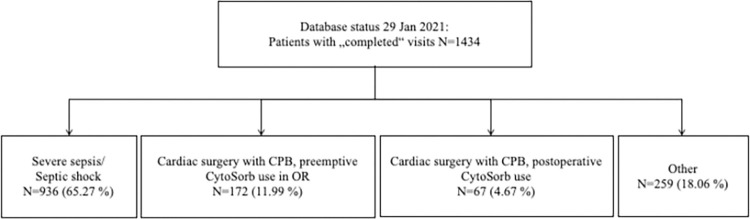
The number of patients by indication. CBP, cardio-pulmonary bypass, OR operating room.

From the beginning of the Registry (18 May 2015) until the 29 January 2021, 1434 patients were entered from by 46 centres, 19 of which were university hospitals, 18 academic teaching hospitals and 9 general or acute care hospitals. The number of patients treated by different indications is summarized in the flow-chart (Fig 1).

### Whole cohort

Demographic, baseline and treatment characteristics are summarized in Table 1, which also indicates the missing data in the “N” columns. 88.4% of patients received up to 5 treatments, including 43.7% who received only one single treatment. CytoSorb was applied together with renal replacement therapy (RRT) in 96% of the treatments. Further details are depicted in Table 1.

**Table 1:** Demographics, treatment characteristics and baseline parameters.

Parameter	Sepsis / septic shock		Cardiac surgery with CPB—preemptive		Cardiac surgery with CPB—postoperative		Other indication		Total	
	Mean±Std; median[IQR]	N (936)	Mean±Std; median[IQR]	N (172)	Mean±Std; median[IQR]	N (67)	Mean±Std, median[IQR]	N (259)	Mean±Std, median[IQR]	N (1434)
**Age [years]**	62.2 ± 14.3	936	61.0 ± 13.5	172	64.7 ± 12.5	67	54.7 ± 16.4	259	60.8 ± 14.8	1434
**Male/Female**	610/326	936	126/46	172	59/8	67	165/94	259	960/474	1434
**APACHE II score**	28.2 ± 8.6	811	N.A.	N.A.	25.5 ± 8.2	60	24.1 ± 10.0	213	27.2 ± 9.0	1084
**Predicted mortality [%]**	66.4 ± 22.5	811	N.A.	N.A.	42.5 ± 25.0	60	50.8 ± 27.2	213	62.0 ± 24.8	1084
**SOFA score**	14.3 ± 3.8	805	9.6 ± 3.3	119	14.7 ± 3.0	63	13.2 ± 4.8	217	13.7 ± 4.1	1204
**Number of adsorbers**	2 [1–39]	931	1 [1–9]	172	1 [1–11]	67	2 [1–25]	257	2 [1–39]	1427
**Total duration of treatment (h)**	43 [0.3–792]	931	2.9 [1–169]	172	38.8 [2.8–234]	67	47.4 [0.7–484]	257	37.7 [0.3–792]	1427
**Treatment time per adsorber (h)**	20 [0.1–105]	3329	3 [0.8–72]	202	24 [0.5–78]	133	24 [0.2–267]	678	20 [0.1–267]	4342
**Time between treatments (h)**	2.6 [0.1–7.7]	2398	2.9 [0.8–19.2]	30	0.8 [0.0.-5.6]	66	0.17 [0.0–8.0]	421	2.3 [0.1–7.7]	2915
**Blood pump flow rate (mL/min)**	150 [130–180]	3327	300 [180–400]	202	120 [100–160]	133	140 [100–160]	678	150 [120–180]	4340
**Combined with RRT, n [%]**	3216 [97]	3323	60 [30]	201	122 [92]	133	642 [96]	672	4040 [93]	4329
**HCO**_**3**_ **–min (mmol/L)**	18.6 ± 5.1	836	NA	NA	18.7 ± 3.8	64	19.2 ± 5.5	245	18.7 ± 5.1	1145
**Creatinine–max (mg/dL)**	2.4 ± 1.4	383	1.5 ± 0.7	28	2.0 ± 0.7	42	2.7 ± 2.1	88	2.3 ± 1.5	541
**Blood urea nitrogen (pg/mL)**	15.2 ± 10.5	896	NA	NA	12.0 ± 7.1	67	14.3 ± 10.8	250	14.8 ± 10.5	1213
**Total bilirubin (mg/dL)**	1.6 [0.8–3.5]	849	0.7 [0.4–1.0]	145	1.6 [1.0–2.3]	66	2.8 [1.0–11]	246	1.5 [0.7–3.6]	1306
**Leukocytes–min (G/L)**	13.4 ± 11.3	929	NA	NA	13.1 ± 8.3	66	13.9 ± 9.0	253	13.5 ± 10.7	1248
**Leukocytes–max (G/L)**	18.2 ± 17.6	588	NA	NA	19.8 ± 8.8	49	18.6 ± 10.7	101	18.3 ± 16.33	738
**Platelets–min (G/L)**	150.1 ± 111.5	928	222.8 ± 91.0	163	126.6 ± 55.5	67	131.8 ± 92.6	254	154.1 ± 107.2	1412
**Platelets–max (G/L)**	181.5 ± 115.9	582	312.3 ± 339.3	29	165.8 ± 64.9	49	178.9 ± 121.2	104	185.1 ± 131.8	764
**CRP at T1 (mg/L) (Mean ± Std [Range])**	179.6 ± 136.5 [0.3–1200]	866	50.4 ± 67.2 [0.1–300]	161	71.0 ± 90.1 [0.4–521]	67	86.8 ± 104 [0–495]	219	142.8 ± 133.3 [0–1200]	1313
**PCT at T1 (ng/mL) (Mean ± Std [Range])**	34.9 ± 70.9 [0–995]	765	9.0 ± 39.1 [0–222]	32	19.1 ± 27.3 [0.1–139]	52	13.9 ± 30.4 [0.1–179]	161	29.9 ± 64.1 [0–995]	1010
**IL-6 at T1 (pg/mL) (Median [Range])**	4240 [0->10^7^]	308	23 [2–5000]	71	446 [69–5000]	41	592 [0->10^8^]	69	1034 [0->10^8^]	489

ICU, intensive care unit; LOS, length of stay; MV, mechanical ventilation

Data are presented as mean ± standard deviation, mean [95% CI, confidence intervals], median [interquartile ranges] as appropriate.

IL-6 shows lognormal distribution, transformation ln(value+1) was used for analysis, hence geometric mean of ratio with 95% confidence interval is given.

**Fig 2:**
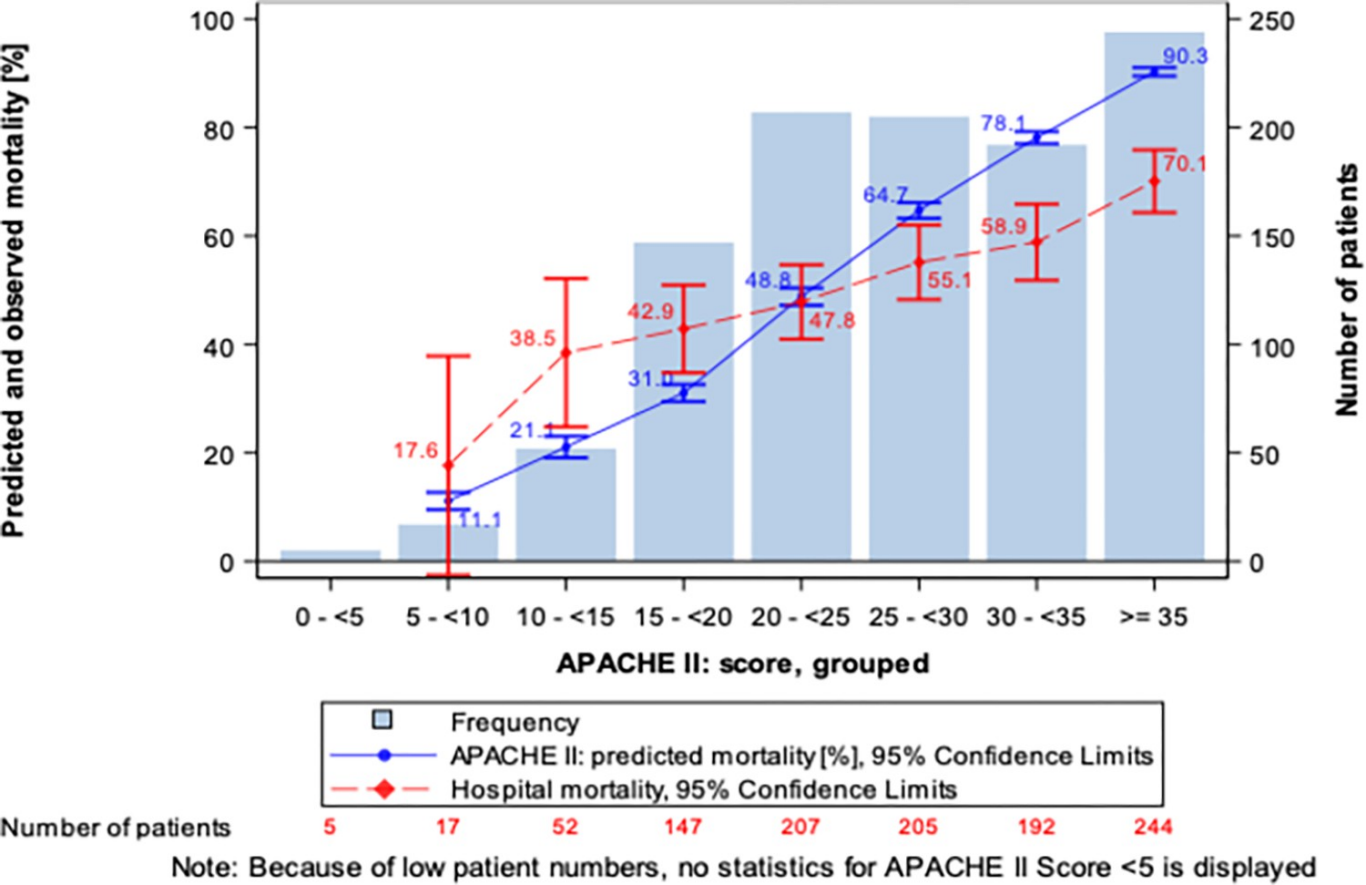
Actual and predicted mortality in the whole sample. ITT, number of patients. See text for explanation.

Actual mortality in the ICU was 47.8%, and hospital mortality was 50.1%. The primary outcome was the difference between the predicted and observed mortality that is summarized in detail in Fig 2. Overall, there was no significant difference between the predicted and actual mortality. Observed mortality was significantly higher as compared to predicted in the APACHE II range of 15–20, but it was significantly lower when the APACHE II was 30 or greater.

Characteristics of organ support are summarized in Table 2. Overall SOFA scores did not change significantly between T1 and T2. However, both the cardiovascular and the pulmonary subscores showed significant differences by T2 (Table 2).

**Table 2:** Outcome parameters.

Parameter	Sepsis / septic shock		Cardiac surgery with CPB–preemptive		Cardiac surgery with CPB–postoperative		Other indication		Total	
	N_total_		N_total_		N_total_		N_total_		N_total_	
Mortality at the end of CytoSorb therapy, n[%]	182 [19]	936	2 [0.1]	170	4 [6]	63	28 [11]	259	216 [15]	1434
ICU mortality, n[%]	524 [57]	928	17 [10]	170	17 [26]	66	121 [47]	256	679 [48]	1420
Hospital mortality, n[%]	548 [59]	923	18 [11]	168	17 [26]	66	129 [50]	253	712 [50]	1410
LOS ICU–survivors (days)	37.1 ± 34.0 [16–44]	400	8.1 ± 12.7 [3–8.5]	152	23.1 ± 24.5 [10–20]	49	25.4 ± 25.1 [10–34]	135	25.4 ± 25.1 [9–36]	736
LOS ICU–non-survivors (days)	19.7 ± 24.9 [4–25]	522	8.2 ± 11.5 [2–8]	17	15.5 ± 13.4 [8–23]	17	14.9 ± 15.5 [4–20]	119	18.4 ± 23.2 [4–23]	675
MV–survivors (days), median [IQR]	19 [7.5–32]	392	2 [1–3]	153	7 [5–17]	49	6 [1–19]	135	9 [2–26]	729
MV–non-survivors (days), median[IQR]	10 [3–20]	515	3 [2–7]	17	8 [4–14]	17	8 [3–16]	119	9 [3–18.5]	668
RRT–survivors (days), median[IQR]	9.5 [4–20]	392	0 [0–0]	149	7 [4–11]	49	7 [3–14]	135	6 [2–15]	725
RRT–non-survivors (days), median[IQR]	5 [2–13]	513	3 [1–5]	17	6 [4–10]	17	8 [3–12]	117	6 [2–13]	664
Days on vasopressors–survivors, median[IQR]	15 [6–29]	390	2 [1–3]	150	5 [4–14]	45	5 [1–12]	133	8 [3–20]	718
Days on vasopressors–non-survivors,median[IQR]	9 [3–18]	511	3 [2–7]	17	8 [5–15]	15	6 [3–12]	118	8 [3–17]	661
Change in SOFA score (T2-T1), mean[CI]	0.13 [-0.2, 0.4]	537 179†	0.6 [-0.03,1.3]*	111 1†	0.96 [0.03, 1.9]	56 4 †	0.05 [-0.4, 0.5]	172 28 †	0.23 [0, 0.5]*	876 212 †
Change in CVS subscore (T2-T1), mean[CI]	-0.54 [-0.6,-0.5]*	717	-0.05 [-0.4, 0.3]	146	-0.5 [-0.8, -0.17] *	62	-0.3 [-0.5, -0.09] *	221	-0.43 [-0.5,-0.3]*	1146
Change in pulmonary subscore (T2-T1), mean[CI]	-0.35 [-0.4,-0.3]*	662	0.18 [-0.05, 0.4]	142	-0.14 [-0.4, 0.2]	58	-0.07 [-0.2, 0.07]	206	-0.21 [-0.3,-0.2]*	1068
Delta CRP (T2-T1) (mg/L), mean[CI]	-46.4 [-57.5,-35.3]*	585	40.1 [26.9, 53.2]*	155	42 [14, 70]*	61	8.5 [-6.8, 23.8]	167	-17.5 [-25.5,-9.5]*	968
Delta PCT (T2-T1) (ng/mL), mean[CI]	-18.2 [-23.6,-12.8]*	488	-6.2 [-28.0,15.6]	22	-4.1 [-11.1, 3.0]	44	-8.8 [- 14.0, -3.5]	99	-15.4 [-19.6,-11.2]*	653
Delta IL-6 (T2/T1) (pg/mL), geometric mean [CI]	-2.6 [-3.0, -2.2]*	163	1.9 [1.3, 2.5]	61	-1.9 [-2.3, -1.4] *	31	-1.2 [-1.9, -0.4] *	32	-1.4 [-1.7, 1.1]	287

ICU, intensive care unit; LOS, length of stay; MV, mechanical ventilation.

Data are presented as mean ± standard deviation, mean [95% CI, confidence intervals], median [interquartile ranges] as appropriate.

† Number of patients who died under hemoadsorption

IL-6 shows lognormal distribution, transformation ln(value+1) was used for analysis, hence geometric mean of ratio with 95% confidence interval is given.

*, p < .05

Basic laboratory data are depicted in Table 1. Regarding inflammatory markers, C-reactive protein (CRP) was measured in most patients (91.6%), procalcitonin (PCT) in 70.4% and interleukin (IL)-6 in 34.1% of patients. Changes of these could only be evaluated in 67.5–45.5–20.2% of cases, respectively. In the whole cohort CRP and PCT decreased significantly from T1 to T2. IL-6 also decreased but it did not reach statistical significance (Table 2).

Regarding the physicians’ subjective assessment about the efficacy of hemoadsorption therapy, overall 53.8% of cases noted improvement, in 30.2% no change and in 4.0% deterioration was reported (Table 3).

**Table 3:** Subjective assessment.

Change due to CytoSorb therapy	Sepsis/septic shock	Cardiac surgery with CPB—preemptive	Cardiac surgery with CPB—postoperative	Other indication	Total
Total number of patients	928	171	66	256	1421
Very much improved, n[%]	95 [10]	6 [4]	6 [9]	35 [14]	142 [10]
Much improved, n[%]	187 [20]	17 [10]	26 [39]	82 [32]	312 [22]
Minimally improved, n[%]	191 [21]	38 [22]	19 [29]	62 [24]	310 [22]
No change, n[%]	292 [32]	89 [52]	7 [11]	41 [16]	429 [30]
Minimally worse, n[%]	4 [0.4]	0 [0]	0 [0]	3 [1]	7 [0.5]
Much worse, n[%]	26 [3]	0 [0]	1 [2]	7 [3]	34 [2]
Very much worse, n[%]	13 [1]	0 [0]	2 [3]	0 [0]	15 [1]
No Assessment, n [%]	120 [13]	21 [12]	5 [8]	26 [10]	172 [12]

### Sepsis group

This is the largest cohort within the Registry with 936 (65.3%) patients. Treatment characteristics are very similar to those of the whole study population (Table 1). Treatment was started after the onset of sepsis within a median of 35.5 [min: 0; max: 720] hours.

**Fig 3:**
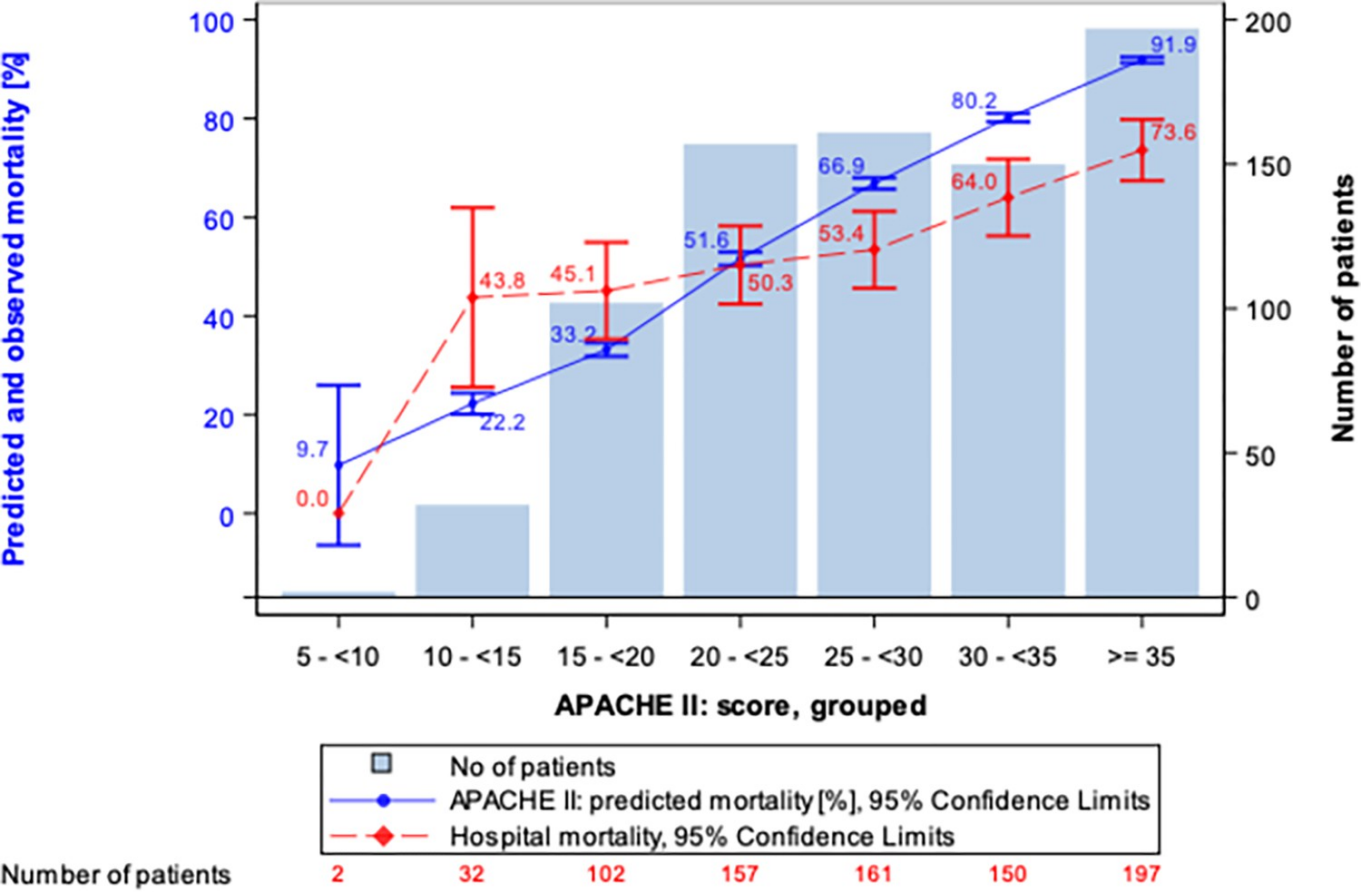
Actual and predicted mortality in the sepsis cohort. ITT, number of patients. See text for explanation.

At the end of hemoadsorption, 80.6% of patients were alive. However, there was no significant difference in the predicted and actual hospital mortality. The relationship between APACHE II predicted mortality and actual mortality was similar and showed similar pattern to that of in the whole cohort (Fig 2, overall, and Fig 3, sepsis cohort). The rest of the outcomes are summarized in Table 2.

At the time of starting hemoadsorption, 83% of patients were receiving norepinephrine, 43.2% dobutamine, 37.1% epinephrine, 40.7% vasopressin, 7.5% dopamine and 48.9% of patients were already on hydrocortisone.

Just as in the whole cohort both the cardiovascular and the pulmonary subscores improved significantly (Table 2). All investigated inflammatory marker levels were higher than in the whole group (Table 1). Changes could be determined for CRP in 67.5%, PCT in 45.5% and IL-6 in 20.0% of patients. CRP and PCT levels decreased significantly by the end of CytoSorb therapy.

### Cardiac surgery

There are two different datasets according to indication in the cardiac surgical Registry: patients who were treated intraoperatively (“Pre-emptive” group, n = 172) and those who received treatment with hemoadsorption after CPB in the ICU postoperatively (Postoperative group, n = 67). The median EUROscore II [IQR] was 5.1 [2.6–14.2] for the pre-emptive and 9.7 [5.0–21.5] for the postoperative patients.

In the pre-emptive group the majority of patients underwent heart valve surgery (n = 137, 79.7%) and/or coronary artery surgery (n = 40, 23.3%). In the postoperative group this distribution was 61.2% and 41.8%, respectively. Due to the nature of intraoperative treatment, it lasted only a few hours as compared to all other cohorts. The rest of the baseline characteristics for both subgroups are summarized in Table 1.

Survival was 98.8% (Pre-emptive group) and 94.0% (Postoperative group) at the end of the therapy. ICU/hospital mortality was 9.9% and 10.5% in the Pre-emptive group (Table 2), and 25.8% in the Postoperative group (Table 1). The rest of the outcomes are depicted in Table 2.

There were just a few specific differences in these groups as compared to the whole cohort. Although there was a tendency of improvement in both cardiovascular SOFA subscore in both groups, it only reached statistical significance in the postoperative group (Table 2).

Similar to the other groups, patients were already on vasopressor support at the start of hemoadsorption. The most frequently applied vasopressors were norepinephrine (pre-emptive group: 73.3%, postoperative group: 78.2%), and epinephrine (53.5% and 51.9%, respectively) and 53.0% and 57.8% patients received hydrocortisone, respectively.

Regarding inflammatory markers, CRP increased significantly in both groups which is exactly the opposite of that seen in the sepsis cohort. In the postoperative group IL-6, which was determined in 46.3% of patients, decreased significantly from T1 to T2 (Table 2).

According to the physicians’ subjective assessment, the least improvement was observed in the Pre-emptive group (35.6%) within the total study population, while in the Postoperative group physicians reported the highest percentage of improvement (77.3%) (Table 3).

## Other indications

The final cohort of patients received CytoSorb therapy due to several different indications (Table 4). Their general demographics and baseline characteristics are summarized in Table 1.

**Table 4:** Indications other than sepsis and cardiac surgery.

Other Indication	No (%) of patients
**Liver failure**	109 (42.1 %)
**Acute pancreatitis**	32 (12.4 %)
**Trauma**	14 (5.4 %)
**ARDS with ECMO**	28 (10.8 %)
**Other indication**	91 (35.1 %)

The general characteristics of this very heterogeneous subgroup are similar to that of the whole cohort.

Their actual mortality was similar to the APACHE-II-predicted, around 50%. Change in SOFA scores also showed significant improvement in cardiovascular subscore. Change of PCT and IL-6 levels were determined in 38.2 and 26.6% of patients respectively and showed significant reduction (Table 2). Myoglobin was also measured in 26 patients in this group which showed a significant reduction between T2-T1: -11,578 [-20,594 to -2,562] μg/L. Serum bilirubin levels were the highest in this group, showing an overall decrease (determined in 201 cases), but it did not reach statistical significance: -1.81 [-2.72;-0.9] mg/L.

Physicians’ satisfaction was also similar to the overall cohort’s with 69.9% reporting improvement.

### Safety issues

The only notable change in routinely measured laboratory parameters during treatment was that the platelet count (minimum value over 24 h) dropped significantly in the total study group and also in all subgroups (other data are only shown at baseline in Table 1). In the whole cohort (n = 1130) it was: -74.2 [-84.7 to -63.7] G/L.

In 1403 patients (97.8%) there were no reported treatment related complications. There were 43 complications that occurred in 31 patients during treatment. Details of these complications are depicted in S1 and S2 Tables in S1 File.

This is the largest report on the features on hemoadsorption therapy to date. The International CytoSorb Registry is a unique initiative aimed to collect information under real-life circumstances via broad scale, centralized, structured and comprehensive documentation of data in order to enhance our knowledge, improve clinical efficacy and optimize its therapeutic application.

Most of the participating centres (80.4%) have academic affiliations. From the 46 study sites, out of the 1434 patients, 1432 provided data for T1, 1427 patients had data on the treatment phase available, 1421 had data for T2 and 1421 had follow-up-data. Undoubtedly, substantial amount of data was missing at T2, especially in the case of inflammatory markers (CRP, PCT, IL-6), but we still ended up with several hundreds of samples to be evaluated.

The fact that most patients were on vasopressors and already on hydrocortisone, plus the high severity scores and the presence of multiple organ failure with more than 4 system failures in the vast majority, underlines that these patients were very sick and most likely received hemoadsorption as an adjunctive rescue therapy in a refractory disease state.

### Primary outcome

In the original protocol of the International Registry the primary outcome was defined as difference between predicted mortality by scoring systems (APACHE II/SAPS II) and actual mortality after intervention during hospital stay (ClinicalTrials.gov, Identifier: NCT02312024), in adherence with recommendations for registries for evaluating outcomes [13].

In the current study predicted mortality was lower than actual mortality in the lower ranges of APACHE II scores (15 to ≤20) and better survival was observed in the sicker patients with high APACHE II scores (≥30). One cannot exclude that this repeated observation is the result of a statistical phenomenon that is termed the ‘regression toward the mean’. This is a phenomenon that arises if a random variable is extreme on its first measurement but closer to the mean or average on its second measurement, and if it is extreme on its second measurement but closer to the average on its first [17].

Recent prospective and retrospective case series [18, 19] and a retrospective propensity score matched study in sepsis/septic shock reported that actual mortality was lower as compared to predicted mortality [20]. In a prospective case series by Friesecke et al., predicted mortality was above 80% while the observed was 55% [17]. In another retrospective case series hospital mortality was 62% as compared to the 92% predicted. In a most recent retrospective study predicted mortality was 74.5% and the observed 47.8% [20]. The results of the registry could not confirm statistically significant benefit in mortality in the overall cohort.

The finding that patients at lower risk seem to have worse outcomes than predicted is more difficult to explain. By and large, patients with high severity scores on admission are definitely sick. However, those who are admitted with lower scores can get worse within hours, but the scoring is usually not repeated within the next 24 hours. In a recent study in ICU patients “admission APACHE II scores” were compared to “worst 24-hour APACHE II scores” [21]. Although the authors found no statistically significant difference between the two scores, but the actual hospital mortality was higher (16%) than predicted by the “admission APACHE II” (12%). The repeated “worse APACHE II” was higher (15%) and closer to the actual hospital mortality. Unfortunately, these were really low risk patients and we do not know if this phenomenon would not be amplified in higher risk patients.

Nevertheless, it is important to note that patients entered into the Registry had very high baseline mortality, in fact APACHE II and SAPS II scores were higher than in any other sepsis trial [21].

### Secondary outcomes

Overall SOFA score changed non-significantly in all cohorts but the pre-emptive cardiosurgical subgroup. However, the cardiovascular subscore of SOFA improved in all subgroups except the pre-emptive cardiac surgical patients. Hemodynamic stabilization has been reported in septic shock [8, 18, 19], cardiac surgery [22, 23] and also in liver failure [24, 25] patients. Our hypothesis-generating results also suggest that hemodynamic stabilization and/or “shock reversal” could be used as primary outcomes in future trials on hemoadsorption.

The pulmonary subscore also improved during the therapy in the total population and also in the Sepsis/Septic shock subgroup. There are very limited data in this field, but two recent case series reported positive result in this regard. Kogelmann et al. evaluated the effects of hemoadsorption in patients on veno-venous ECMO and found by the time hemoadsorption was terminated, PaO_2_/FiO_2_, peak inspiratory pressure and positive end expiratory pressure had improved significantly after one 24 hour treatment and further improved by the end of the full course of therapy [26]. In another case series on 9 patients with septic shock, some improvement of PaO_2_/FiO_2_ and reduced extravascular lung water (EVLW) were observed, but these changes did not achieve statistical significance [27]. Our data on several hundreds of patients provides further support to the abovementioned small case series that their findings on changes in pulmonary function maybe worth investigating in the future.

Our data provides further evidence to previous findings that during hemoadsorption therapy inflammatory marker levels, such as PCT and IL-6 are significantly reduced. One of the main messages of the first randomized controlled clinical trial by Schaedler et al. in patients with septic shock was, that even within a short treatment period of 6 hours and in patients with relatively low IL-6 values, levels were found to decrease [28]. A more recent randomized, controlled pilot study showed that PCT was also removed extremely effectively in patients with septic shock [10], confirming the findings of previous case series [18]. Therefore, the elimination of these two biomarkers by hemoadsorption may be worthwhile to assess in future studies.

It is important to note that physicians terminated therapy after a few—if not only one–treatments, although patients stayed in the ICU for a median of 11 (non-survivors) to 19 days (survivors). This could be explained by the substantial clinical improvement presented in this analysis, namely, that the improvement in cardiovascular and pulmonary subscores, plus the decreasing biomarker levels and the very high survival rate at T2, leading to the high grade of subjective observation of improvement by the physicians which led to them to decide to end the therapy early.

### Different indications sepsis/septic shock

This was the largest cohort, meaning that physicians still see this indication as the most important. Based on the current results of the Registry, the most likely population who may benefit the most are patients with refractory septic shock and especially those in whom there is also an indication for RRT. It is important to note that the patients in this cohort of the registry were extremely sick. A systematic review including 166 479 patients from 92 sepsis studies by Shankar-Hari et al. [29] and a more recent meta-analysis by Vincent et al. [30], reported crude mortality of 46.5%, and hospital mortality of 38%, respectively. In our cohort APACHE II predicted mortality was 66% and the actual mortality of 59%, substantially higher than in any other septic shock trial so far. This indicates the challenge of the selection of the right patient population for future trials, who are sick enough, but not too sick to benefit the most from blood purification.

#### Cardiac surgery

It has been well documented that CPB induces inflammatory response that can potentially cause postoperative organ dysfunction [31, 32]. Therefore, it is not surprising that almost 3 times more patients received CytoSorb in a pre-emptive fashion than postoperatively. However, the least improvement was observed by physicians and also supported by clinical data in the pre-emptive group. This is in accord with the results of 3 small randomized clinical trials performed in the last few years, in which hemoadsorption was applied without clear outcome benefit [33–35]. However, the common feature of these trials is that they included patients with similar severity and pathology to that of seen in the Registry with a EuroSCORE of 5.4 [33], 6.1 [34] and 5.1 [35]. On the contrary, when hemoadsorption was applied in patients with infective endocarditis [22, 23] - where the EuroSCORE was 11 [22] and 33.8 [23], aortic surgery [36] and in heart transplantation [37], the authors reported both clinical improvement, and attenuation if the inflammatory response [22, 37]. These results indicate that careful selection of patients scheduled for cardiac surgery with the abovementioned indications are worthwhile to be investigated in randomized trials as they are the most likely to benefit from hemoadsorption therapy.

#### Other indications

It is interesting to note that CytoSorb has been applied in several other indications, such as listed in Table 4. There are successful reports in liver failure [38], pancreatitis [39], rhabdomyolysis [40], patients with drug overdose [41] or ticagrelor and rivaroxaban removal before acute cardiac surgery [42] and hemophagocytic lymphohistocytosis [43]. Adding the results of the current Registry provide further support to those who would like to discover the efficacy of CytoSorb therapy in these and also other unknown territories.

#### Safety issues

In line with all papers published so far, regardless of the type of the study or case report, the 11^th^ analysis of the Registry data further suggest that CytoSorb therapy is safe. However, the Registry is unable to answer all issues concerning safety, including changes in platelet count, removal of certain drugs, etc., questions that have to be answered in future randomized trials.

#### Strengths and weaknesses

Medical registries are important sources of information for quality assurance, for optimized treatment, and they are indispensable for transferring scientific results into clinical routine. We are not aware of any clinical registry in intensive care medicine, which reports such high patient numbers as the current International Registry. Furthermore, the Registry provides useful data for clinical practice, and also for those who are planning to undertake clinical trials with hemoadsorption.

However, it has several limitations. Although centres are encouraged to do so, there is no evidence that every single patient treated with CytoSorb is indeed entered from the sites that have signed up with the coordinating centre in Jena, Germany. Therefore, some selection bias cannot be excluded. The absence of a control groups also adds to the limitations of the results. Furthermore, there was substantial amount of missing data especially at T2, which limits the strength of the secondary endpoints.

Unfortunately, we cannot answer important questions such as the reasons for not starting hemoadsorption in some patients, or how other patients were selected to be treated. This certainly limits the external validity of our findings.

Although the project has been supported financially by CytoSorbents, this was only to cover administrative costs for the management of the registry and was not used for personal payments to encourage recruitment or provide funding for human resources. The lack of personnel could well be one of the main reasons why “only” 46 and mainly academic centres were able and willing to participate.

Another limitation of the study is that the Registry was designed using the consensus criteria [12], which has since become obsolete and didn’t include lactate levels. Therefore, when interpreting the changes in cardiovascular effects this has to be taken into account. Nevertheless, one of the major strengths of this Registry is that it is voluntary, mirrors real-life circumstances and practices, and the data has consistently been of excellent quality since the last publication [11].

## Conclusions

This article summarizes the results of systematic data collection on the largest case series of patients to date treated with extracorporeal cytokine adsorption with CytoSorb. There was no significant difference in the primary outcome of mortality, but there was improvement in cardiovascular and pulmonary SOFA scores and reduction in PCT, CRP and IL-6 levels. Whether these effects translate into overall outcome benefit has to be answered by randomized trials.

## Supporting information

S1 File (DOCX)

## Abbreviations

APACHE II: Acute Physiology And Chronic Health Evaluation II
CBP: Cardiopulmonary bypass
CRP: C-reactive protein
ECMO: Extracorporeal membrane oxygenation
eCRF: electronic case report form
EUROSCORE: European System for Cardiac Operative Risk Evaluation
EVLW: Extravascular lung water
ICU: Intensive Care Unit
IL-6: Interleukin-6
IL-8: Interleukin-8
PCT: Procalcitonin
SAPS: Simplified Acute Physiology Score
SOFA: Sequential Organ Failure Assessment
