# Supplementary material for: Hemoadsorption in the critically ill—Final results of the International CytoSorb Registry
Source: PLoS One. 2022 Oct 25;17(10):e0274315. doi: 10.1371/journal.pone.0274315 (PMC9595535; doi:10.1371/journal.pone.0274315)
Supplement: S1 File — (DOCX) [file pone.0274315.s001.docx]

**Supplementary Data**

**Safety Analysis – Adverse Events**

There is no systematic recording of adverse effects in the registry. 22 complications have been recorded in 15 patients by systematic request by organ/system, 6 cases related to organ system “heart/circulation”, 4 in “respiration”, 3 cases with respect to “blood” and 2 each in “kidneys/excretion”, “nervous system” and “digestive system (incl. liver)”. 3 events reported elsewhere have been repeated as complication with respect to “other” organ/system.

Supplementary table S1: Treatment related complications: complications by organ/system, N= 930

| **Complications (organ/system)** | **No (%) Patients** |
| --- | --- |
| 0 Complications (organ/system) | 910 ( 97.8 %) |
| Blood | 3 ( 0.3 %) |
| Heart/circulation | 6 ( 0.6 %) |
| Kidneys/excretion | 2 ( 0.2 %) |
| Nervous system | 2 ( 0.2 %) |
| Respiration | 4 ( 0.4 %) |
| Digestive system (incl. liver) | 2 ( 0.2 %) |
| Others | 3 ( 0.3 %) |

Supplementary table S2: Treatment related complications: Listing of descriptions, where complications described

|  | **Indication** | **Description** | **Organ/System** |
| --- | --- | --- | --- |
| 1. | Other | Increasing thrombocytopenia and Hb-decrease | Blood |
| 2. | Sepsis / septic shock | Legionella pneumophilia positive result during Cytosorb treatment. Not known if related to Cytosorb! | Respiration |
| 3. | Sepsis / septic shock | Suspected intracranial bleeding and death under heparin anticoagulation | Blood |
| 4. | Other | Thrombocytopenia | Blood |
| 5. | Sepsis / septic shock | Cardiogenic shock | Heart/circulation |
| 6. | Sepsis / septic shock | Torsades de pointes | Heart/circulation |
| 7. | Sepsis / septic shock | Death | others |
| 8. | Other | Rhabdomyolysis | Kidneys/excretion |
| 9. | Sepsis / septic shock | Patient died due to multi-organ failure not responding to therapy | Heart/circulation |
| 10. | Sepsis / septic shock | Acute kidney failure | Heart/circulation |
|  |  | Ischemia legs and arms due to use of ECCOR + inotropes + thrombosis | Heart/circulation |
| 11. | Sepsis / septic shock | Shock liver and hypoglycemia | Digestive system(incl. liver) |
| 12. | Sepsis / septic shock | Acute cholecystitis | Respiration |
|  |  | Severe hemoptoe | Respiration |
| 13. | Sepsis / septic shock | Patient died during Cytosorb dialysis due to worsening septic shock | Heart/circulation |
| 14. | Other | Patient had maximal compromised Psyche. (Psychotic) | Nervous system |
| 15. | Sepsis / septic shock | Patient died during therapy | Heart/circulation |
| 16. | Other | Severe Liver dysfunction - no transplantation according to the transplant center | Digestive system(incl. liver) |
| 17. | Cardiac surgery with CPB preemptive | Hemiparesis right side | Nervous system |
| 18. | Cardiac surgery with CPB preemptive | Acute kidney failure | Kidneys/excretion |
| 19. | Cardiac surgery with CPB preemptive | Limited gas exchange | Respiration |
| 20. | Sepsis / septic shock | ECMO for 18h and 10h. | Respiration |
